# Changes in Lower-Extremity Gait Biomechanics Following High-Cadence Cycling

**DOI:** 10.3390/sports12060159

**Published:** 2024-06-07

**Authors:** Tanner A. Thorsen, Rials J. Hester, Christopher J. Keating

**Affiliations:** 1 School of Kinesiology and Nutrition, The University of Southern Mississippi, Hattiesburg, MS 39406, USA; 2 Facultad de Deportes, Universidad Católica San Antonio de Murcia, Guadalupe de Maciascoque, 30107 Murcia, Spain; cjames@ucam.edu

**Keywords:** gait velocity, gait analysis, cycling, kinematics, kinetics

## Abstract

We sought to investigate the lower-extremity biomechanics underlying increased gait velocity following high-cadence cycling. Ground reaction forces (GRF) and lower-extremity kinematics and kinetics were recorded as 15 healthy adults walked at a self-selected pace prior to and immediately following a 15 min bout of cycling at a cadence of 75 rotations per minute. Propulsive GRF and stance-phase peak dorsiflexion and knee extension angles increased, while peak plantarflexion and hip extension angles decreased. Swing-phase peak dorsiflexion, plantarflexion, knee flexion, and hip flexion angles increased, while peak knee extension angle decreased. Peak dorsiflexion, knee extension, and hip extension angular velocity also increased during swing. No changes in peak joint moments were observed; however, peak positive ankle, knee, and hip joint power generation increased following cycling. Completing high-cadence cycling improves gait velocity by increasing propulsive GRF; increasing joint angular velocity during the swing phase of gait for the ankle, knee, and hip; and increasing positive power production by the ankle, knee, and hip during the stance phase. Increased gait velocity post cycling exercise did not increase lower-extremity joint moments. Cycling may be a viable exercise-based modality for increasing gait velocity, especially in populations where gait ability or joint loading is of particular concern.

## 1. Introduction

Gait velocity is considered an important metric of functional ability and has been extensively studied across the lifespan. Some have even referred to it as the “sixth” or functional vital sign [1,2]. Reduced gait velocity has been associated with numerous health complications from orthopedic trauma and arthroplasty [3] to frailty [4] and metabolic disorder [5,6], and improvement in gait velocity has been the target of many interventions [7].

A substantial body of literature has investigated the biomechanics of walking at faster speeds [1]. In younger adults, spatiotemporal gait parameters (e.g., cadence, step- length, and stride length) have been shown to increase with faster walking speed [8,9]. Small yet significant increases in peak hip flexion, hip extension, knee flexion, and ankle plantarflexion angles have also been shown as walking speed increases [10,11,12]. Walking at faster speeds has also been shown to alter joint kinetics, with plantarflexion moment, knee extension moment, and hip extension moment all demonstrating large effects sizes, suggesting increased joint moments with faster walking speed [10,11]. Finally, due to increased gait velocity, first and second peak vertical ground reaction force (GRF), the anteriorly directed component of the ground reaction force vector (propulsive GRF), and the posteriorly directed component of the GRF vector (braking GRF) all increased with gait velocity [13,14,15].

We recently demonstrated that performing single bouts of cycling at a pedaling cadence higher than the typical gait cadence increased self-selected gait velocity during level over-ground walking [16]. We compared self-selected gait velocity immediately before and after a bout of cycling between three groups: one group that cycled at a comparable cadence to typical gait cadence of 55 revolutions per minute (RPM) but at an increased work rate of 1.5 Watts per kilogram (W/kg), one group that cycled at 75 RPM (a higher cadence than walking) at a normalized work rate of 1.0 W/kg, and a control group who cycled at a comparable cadence to typical gait cadence of 55 RPM at a normalized work rate of 1.0 W/kg. In the group who cycled at a higher cadence, post-cycling self-selected gait velocity increased by 8.5% from 1.19 m/s to 1.29 m/s. Gait velocity for the other groups remained constant between conditions. One of the primary mechanisms responsible for the increase in gait velocity was an increase in gait cadence, manifested through reduced stance and swing-phase times, and not adaptations of other spatiotemporal parameters of gait such as step length or step width.

Notably, our previous work measured self-selected gait velocity following a cycling intervention and not a prescribed, experimental, or forced increase in gait velocity. Cycling at cadences higher than the typical gait cadence appears to improve self-selected gait velocity, at least in the short term. However, the biomechanical changes in the lower extremity associated with this improvement in velocity remain unclear. Determining the gait adaptions that facilitate this increased gait velocity can provide greater insight into the mechanisms underlying increased gait velocity and be influential for application in settings where improving gait velocity may be beneficial (e.g., rehabilitation, sports performance, fall prevention, etc.).

Therefore, we sought to investigate the GRF and sagittal plane joint kinematics and kinetics underlying the increase in gait velocity consequential of increased gait cadence following high-cadence cycling. If increased gait velocity following cycling produces similar biomechanics to those established in the prescribed or forced fast-walking literature, we predicted the following four hypotheses would be supported: (1) an increase in vertical and propulsive GRF; (2) an increase in stance phase peak sagittal plane ankle plantarflexion, knee extension, and hip extension angles, with reduced knee flexion and hip flexion angles; (3) an increase in swing-phase peak sagittal plane ankle plantarflexion, knee extension, and hip extension angles, with reduced knee flexion and hip flexion angles; and (4) increased peak sagittal plane propulsive joint moments, namely plantarflexion, knee extension, and hip flexion moments.

## 2. Materials and Methods

### 2.1. Participants

As part of a larger study, 15 recreationally active young adults were recruited to attend one laboratory visit [16]. Participant demographic information is presented in Table 1. Inclusion criteria included being recreationally active adults between the ages of 18–35, defined as engaging in moderate-to-vigorous activity three days or more per week for a total of 150 min/week or more [17]. Exclusion criteria included having lower-extremity injury within the past 6 months or having any neurological or cardiovascular condition that would affect their ability to walk, ability to ride a stationary cycle ergometer, or participate in cardiovascular exercise. Participants wore tight-fitting spandex shorts and a t-shirt. All participants provided written informed consent, which was approved by the local Institutional Review Board.

### 2.2. Protocol

Participants first completed two ten-meter walk tests (10 MWT) along a 10 m walkway in a motion capture volume equipped with six in-ground in-line force platforms [18]. Each 10 MWT followed standard protocols where participants were instructed to walk at a comfortable (preferred) walking speed down a 10 m walkway straight through a motion capture volume equipped with six in-ground force platforms. Gait biomechanics were captured by recording each 10 MWT through the middle 6 m of the walkway [18], recorded with a two-gate photocell timing system (Blue, Dashr, Lincoln, NE, USA). Following the completion of both 10 MWT, participants were instructed to complete one 15 min bout of cycling at a normalized work rate of 1.0 watt per kilogram (W/kg) at 75 RPM. After the 15 min bout of cycling, two additional post-cycling 10 MWT were performed through the motion capture volume. During both pre- and post-cycling 10 MWT time, kinematics, and kinetics were simultaneously recorded. In order to capture the typical self-selected gait pattern of each participant, no warm-up or recovery periods were completed between walking and cycling conditions.

Participant effort during cycling conditions was monitored by research team members via visual inspection of cycling cadence indicated on the digital display of the cycle ergometer. The normalized work rate of 1.0 W/kg was calculated using The American College of Sports Medicine (ACSM) equations and used participant mass and a work rate of 75 RPM as inputs to the algorithm. As such, participants were instructed to maintain a cadence of 75 ± 2 RPM at all times during the 15 min bout of cycling [16]. All walking and cycling activities were completed in a climate-controlled indoor laboratory.

### 2.3. Instrumentation

A ten-camera motion analysis system (240 Hz; Qualisys, Götenburg Sweden) was used for three-dimensional kinematic data collection. To define anatomic segments, passive reflective anatomic markers were placed bilaterally on the iliac crest, greater trochanter, medial and lateral epicondyles, medial and lateral malleoli, and on the outside of the shoes at the location of the head of the 5th metatarsal, head of the 1st metatarsal, and the 2nd toe. To track each segment, a semi-rigid thermoplastic shell with four retroreflective tracking markers were placed on the shanks, thighs, and pelvis. Four individual retroreflective tracking markers were affixed to the heel counter of each shoe. Prior to data collection, a static standing calibration trial was recorded. All GRF were recorded using six in-ground force plates positioned within the 10 MWT walkway and motion capture volume (1200 Hz, American Mechanical Technology Inc. Watertown, MA, USA).

Before the data collection session began, participants were first fitted to a mechanically braked stationary cycle ergometer (828e, Monark, Vansbro, Sweden). Saddle height was determined by obtaining a knee flexion angle of 25° to 30° when the pedal was placed at the bottom dead center, measured with a handheld goniometer [19,20,21]. The saddle fore/aft position was also adjusted such that the participant’s knee was in line with the pedal spindle when the crank was in the forward horizontal (90°) position, measured by hanging a plumb-bob from the inferior pole of the patella [21]. The handlebar position was set such that the angle between the participant’s trunk and thigh was 90°, as measured by a handheld goniometer when the crank was at 90°.

### 2.4. Data Analysis

Marker trajectories and GRF signals were exported to and all variables of interest were calculated in Visual3D biomechanical analysis suite (Version 6.0, C-Motion; Germantown, MD, USA). Angular computations were completed using a Cardan rotational sequence (X-Y-Z) based on the right-hand rule to define angular kinematic and kinetic variable conventions. Kinematic and GRF data were filtered using a zero-lag fourth-order Butterworth low-pass filter at 6 Hz [22]. Joint moments were computed as internal moments and expressed in the joint coordinate system [23]. Force was normalized to body weight, and joint moments and power variables were normalized to body mass. Peak joint angles and angular velocities were identified during the stance and swing phases of the stride of the right leg. Peak GRF, joint moments, and joint powers were identified during the stance phase of one step of the right foot. The stance phase was defined between heel strike (the first time point vertical GRF ascended above a force platform threshold of 10 N) and toe-off (defined as the first time point vertical GRF descended below a force platform threshold of 10 N) of the right limb, whereas the swing phase was defined from heel-strike of the right foot to the subsequent heel strike of the right foot [24].

### 2.5. Statistical Analysis

Primary variables of interest included three-dimensional GRF; peak sagittal plane joint angles of the ankle, knee, and hip; and peak sagittal plane plantarflexion and dorsiflexion, knee flexion and extension, and hip flexion and extension joint moments. We also included the ankle, knee, and hip joint angular velocities and joint powers as well as secondary, explanatory variables. Given the symmetrical nature of human gait, we analyzed all variables from the right limb, which was also the self-reported dominant limb of all participants. Limb dominance was assessed as the limb used when kicking a ball [25].

Paired-samples *t*-tests were conducted to detect differences between pre- and post-cycling variables (Version 27.0; SPSS, IBM, Armonk, NY, USA). An α level of 0.05 was set a priori, and Cohen’s d was used as an estimate of effect size, using Cohen’s definitions of small, 0.2; medium, 0.5; and large, 0.8 effect for interpretation [26].

## 3. Results

The current data indicate that high-cadence cycling increased gait velocity through a multifaceted mechanism. Notably, the increased gait velocity was characterized by a reduction in stance time and an increase in cadence. These effects are underpinned by heightened propulsive GRF and increased angular velocity of the ankle, knee, and hip joints during the swing phase of gait.

**Figure 1:**
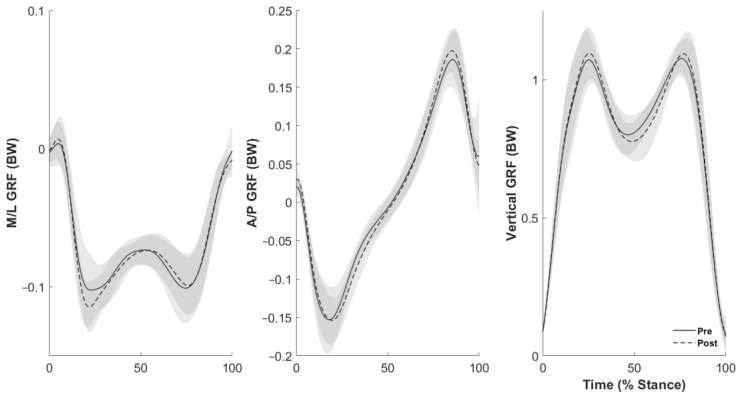
Ground reaction forces (GRF) during the stance phase of gait. Solid black (pre-cycling) and dashed (post-cycling) line indicate mean values, with the gray shaded regions representing ± 1 standard deviation. Top panel: mediolateral directed (M/L) GRF. Middle panel: anteroposterior directed (A/P) GRF. Bottom panel: vertically directed GRF.

Peak propulsive GRF increased following the cycling exercise (*p* < 0.05, Table 2, Figure 1). There were no other changes in mediolateral or vertical GRF between conditions (Table 2).

**Figure 2:**
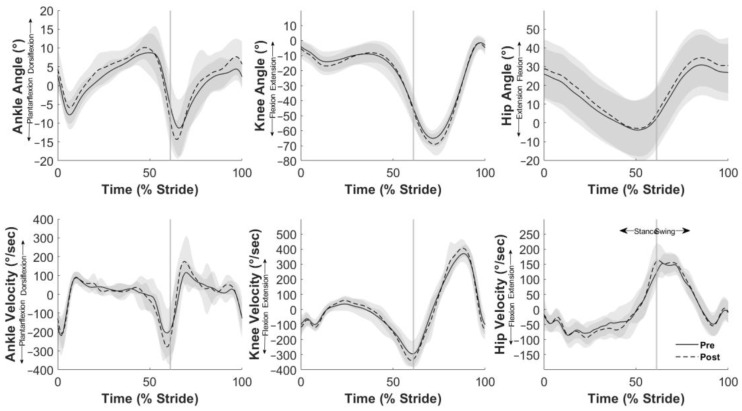
Joint angles (top row) and velocities (bottom row) of the ankle, knee, and hip joint during one stride of gait. Solid black (pre-cycling) and dashed (post-cycling) line indicate mean values, with the gray shaded regions representing ± 1 standard deviation. The vertical gray line indicates toe-off, demarcating stance and swing phases. Positive rotations include ankle dorsiflexion, knee extension, and hip flexion.

During stance, the peak dorsiflexion angle increased (*p* = 0.024, Table 3) and peak plantarflexion angle decreased (*p* = 0.05, Table 3); however, there was no change to peak ankle dorsiflexion velocity (Table 3, Figure 2). Following cycling, participants demonstrated increased knee flexion angle (*p* = 0.012, Table 3) and increased knee extension angular velocity (*p* = 0.007, Table 3). The peak hip extension angle during stance was also reduced post-cycling (*p* = 0.036, Table 3).

During the swing phase, the peak ankle dorsiflexion angle, plantarflexion angle, and dorsiflexion velocity all increased (all *p* < 0.012, Table 3) following cycling. Participants walked with reduced peak knee extension (*p* = 0.033, Table 3), increased peak knee flexion (*p* = 0.012, Table 3), and increased knee extension velocity (*p* = 0.008, Table 3). Increased peak hip flexion angle (*p* = 0.005, Table 3) and peak hip flexion velocity (*p* = 0.003, Table 3) were also observed following cycling.

**Figure 3:**
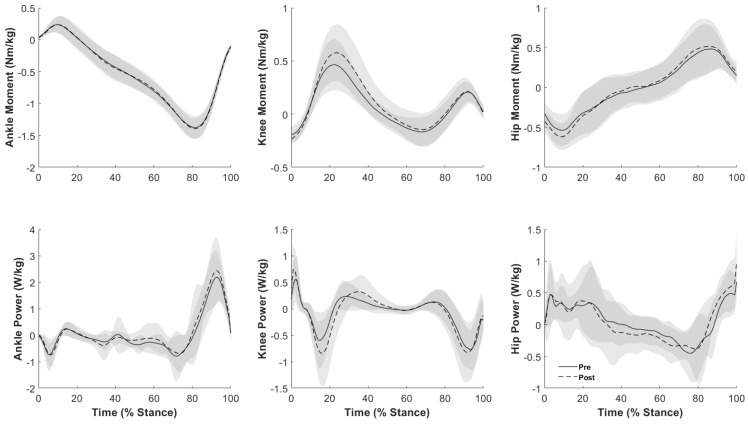
Joint moments (top row) and powers (bottom row) of the ankle, knee, and hip joint during the stance phase of gait. Solid black (pre-cycling) and dashed (post-cycling) line indicate mean values, with the gray shaded regions representing ± 1 standard deviation.

No significant differences were observed for any joint moments (Table 4). Peak positive joint power generation was increased at the ankle (*p* = 0.001. Table 4, Figure 3), knee (*p* = 0.044, Table 4, Figure 3), and hip (*p* = 0.026, Table 4, Figure 3). Peak negative power absorption at the hip was greater following cycling (*p* = 0.020, Table 4, Figure 3).

## 4. Discussion

We sought to investigate GRF and the sagittal plane joint kinematics and kinetics underlying the increase in gait velocity consequential of increased gait cadence following high-cadence cycling.

We first hypothesized an increase in vertical and propulsive GRF following cycling exercise; however, only the propulsive GRF was greater (Figure 1). The rationale for this hypothesis stems from previous research that identified differences in vertical and propulsive GRF [13,14,15]. When walking, propulsive GRF accelerates the center of mass in the direction of travel; thus, increased propulsive GRF (9% increase in this case) with increased velocity is expected. Comparable vertical GRF before and after cycling in this study is likely due to the independence of gravity on walking speed and a reflection on the young/healthy nature of participants to better control vertical accelerations of their center of mass during walking [27].

Our second and third hypotheses investigated stance- and swing phase-specific kinematics. The key findings of this research are the notably different joint kinematics before and after cycling between the stance and swing phases of the gait cycle, partially supporting our second hypothesis and fully supporting our third hypothesis. During stance, the peak ankle and knee joint angles appear to be shifted towards a more dorsiflexed position at the ankle and a more flexed position at the knee—however, the joint range of motion (ROM) was similar. During the swing phase, participants demonstrated an ankle ROM that was shifted toward a reduction in dorsiflexion but with comparable ankle joint and knee joint ranges of motion. Perhaps more importantly, participants performed ankle and knee joint excursions during stance and swing with a significantly greater joint angular velocity after cycling. These comparable joint excursions and increased joint velocities likely explain the previously reported reductions in step and stance times [15]. Swing-phase joint kinematics, especially joint angular velocities, play a significant role in increasing gait cadence and subsequently increased gait velocity. In the context of improving walking ability in older adults, Brach and VanSwearingen (2013) suggested that traditional exercise intervention strategies are often aimed at “building a bigger engine” by maximizing performance, (i.e., increasing strength and ROM, enhancing delivery and extraction of oxygen, etc.) [28]. They also suggested that a more appropriate strategy might be to “build a better engine, run more efficiently, and optimize performance”. To accomplish this, they suggested, in part, realigning biomechanical and neuromotor control. We suggest that targeting swing-phase joint kinematics, especially joint angular velocities, through high-cadence cycling, could be one mechanism in which gait performance is optimized to facilitate increased velocity following cycling exercise.

In contrast to prescribed or forced increases in gait velocity, high-velocity power training during cycling may promote an increased self-selected gait velocity [29]. Cycling has been identified as an important low-force high-velocity exercise [7,29]. In this context, Bellumori et al. (2017) investigated the effects of a high-speed cycling intervention on mobility, perceived health, and function of older adults approaching frailty. Although gait velocity was not explicitly tested, rapid neural adaptations were suggested to explain improvements in whole-body mobility, including metrics of gait performance such as the timed up-and-go test [7]. Completing high-cadence cycling exposes participants to vigorous neural activation and contributes to improvements in strength, speed, and balance [7]. It could be argued that increasing the resistance (or work rate) is sensible for exercise progression, but it should also be noted that cycling for 15 min at a cadence of 75 RPM produces approximately 1125 rapid muscle contractions involving the entire lower-extremity musculature, which would be infeasible to achieve through many other exercise modalities [7]. Pairing low-force high-velocity cycling exercise, which is not too physiologically demanding for healthy adults, with a high-quantity set of rapid muscle contractions provides the necessary motor plan training to improve walking function, furthering our ability to optimize walking performance and “build a better engine”, as suggested by Brach and VanSwearingen [28].

Our fourth hypothesis suggested an increase in propulsive joint moments during stance and was not supported by our results. Many studies have reported increased propulsive joint moments (i.e., ankle plantarflexion, knee extension, and hip extension) during faster-paced walking [10,11]; however, the results of our investigation indicate no changes in joint moments following high-cadence cycling. This suggests that the observed effect of increased gait velocity following cycling does not occur at the cost of increased loading across lower-extremity joints. This further supports our conclusion that increased gait velocity is highly influenced by swing-phase kinematics. This is similar to the results reported by Fang et al. (2016), who reported that cycling at higher cadences did not increase the knee extension joint moment, whereas cycling at higher work rates did [30]. If improvements in gait cadence and velocity are heavily influenced through swing-phase joint kinematics and less influenced through stance phase joint mechanics, then consistent joint moments before and after cycling exercise are to be expected. Thus, utilizing cycling exercise as a modality to increase gait velocity may not come with the expense of added load across joints. This may be particularly promising for populations in which increased joint loading may be a primary concern.

As the product of joint moment and angular velocity, joint power has been utilized as a useful predictor of muscle function during gait [31]. Following the completion of cycling exercise, peak positive stance-phase joint power generation increased at the ankle, knee, and hip (Table 4), which aligns with the established body of literature investigating joint kinetics during fast-paced walking [31,32,33]. It has been well understood that many biomechanical parameters, including joint powers, scale with speed [34,35]. Lelas et al. (2003), for example, showed that peak lower-extremity joint power generation systematically increased with walking speed [35]. In addition, the relative contribution of peak positive power generation from each joint in this study remained similar between conditions, suggesting that in our sample of younger adults, propulsive power strategy was still ankle-dominant and did not shift to proximal joints based on speed.

The results of this study should be considered in the context of its notable limitations. First, as this was an analysis of a sample that was part of a larger study, the statistical power may have been reduced for some variables. Second, this convenience sample of younger adults makes generalization difficult for populations in which gait velocity is meaningful, and readers are encouraged to interpret these results with caution. Our primary research focus pertained to the lower-extremity biomechanics prior to and after cycling. As such, we did not record upper-body or trunk kinematics that may also have varied with gait velocity, thus influencing our outcome measures.

## 5. Conclusions

Completing high-cadence cycling improves gait velocity by decreasing stance time and increasing cadence. This was accomplished through increased propulsive GRF; increased joint angular velocity during the swing phase of gait for the ankle, knee, and hip; as well as increased positive power production by the ankle, knee, and hip during the stance phase of gait. Increased gait velocity post cycling exercise did not increase lower-extremity joint moments. Thus, high-cadence cycling may be an attractive modality in populations where joint loading is of particular concern.

## Figures and Tables

**Table 1:** Demographic information for sample participants reported as mean ± s.d.

	*n*	Height (m)	Mass (kg)	Age (Years)
Male	6	1.75 ± 0.08	75.7 ± 4.6	22.6 ± 2.0
Female	9	1.70 ± 0.07	68.5 ± 7.8	21.7 ± 2.1

**Table 2:** Ground reaction forces (GRF) during the stance phase of gait presented as mean ± s.d. normalized to body weight (BW), with the *p*-value and Cohen’s d effect size (*p* (*d*)). LR, loading response, i.e., first peak; PO, push-off, i.e., second peak vertical GRF. **Bold** indicates statistical significance.

	PRE	POST	*p* (*d*)
Peak anterior (propulsive, BW)	0.20 ± 0.03	0.22 ± 0.02	**0.014 (0.632)**
Peak posterior (braking, BW)	−0.16 ± 0.04	−0.16 ± 0.03	0.383 (0.079)
Peak vertical LR (BW)	1.10 ± 0.11	1.12 ± 0.1	0.249 (0.180)
Peak vertical PO (BW)	1.11 ± 0.06	1.12 ± 0.07	0.107 (0.336)

PRE: pre-cycling; POST: post-cycling.

**Table 3:** Joint angles and velocities during one stride of the right leg presented as mean ± s.d., reported in degrees (°) and degrees per second (°/s), with the *p*-value and Cohen’s d effect size, *p* (*d*). **Bold** indicates statistical significance.

	Stance			Swing		
	PRE	POST	*p* (*d*)	PRE	POST	*p* (*d*)
Peak dorsiflexion angle (°)	10.5 ± 2.5	11.7 ± 3.1	**0.024 (0.562)**	5.4 ± 3.2	7.8 ± 4.6	**0.012 (0.656)**
Peak plantarflexion angle (°)	−11.0 ± 2.8	−9.3 ± 3.5	**0.005 (0.777)**	−15.8 ± 5.4	−19.3 ± 5.1	**<0.001 (0.779)**
Peak dorsiflexion velocity (°/s)	100.9 ± 29.0	101.5 ± 28.0	0.450 (0.033)	160.4 ± 66.4	198.5 ± 66.1	**<0.001 (0.877)**
Peak knee extension angle (°)	−6.5 ± 4.1	−7.8 ± 4.5	0.191 (0.302)	0.80 ± 3.2	−0.62 ± 4.4	**0.033 (0.538)**
Peak knee flexion angle (°)	−42.6 ± 4.8	−44.2 ± 5.5	**0.012 (0.655)**	−66.3 ± 4.8	−69.0 ± 5.5	**0.012 (0.656)**
Peak knee extension velocity (°/s)	53.1 ± 25.3	61.9 ± 27.5	**0.007 (0.727)**	387.5 ± 36.2	417.3 ± 39.6	**0.008 (0.700)**
Peak hip extension angle (°)	−16.9 ± 8.1	−13.6 ± 8.8	**0.036 (0.504)**	-	-	-
Peak hip flexion angle (°)	-	-	-	24.6 ± 10.4	29.1 ± 9.3	**0.005 (0.775)**
Peak hip extension velocity (°/s)	−113.9 ± 14.7	−117.2 ± 22.63	0.180 (0.245)		-	-
Peak hip flexion velocity (°/s)	-	-	-	179.3 ± 18.4	197.7 ± 24.5	**0.003 (0.856)**

Note: Peak hip flexion angle during stance occurred at heel strike, and peak hip extension angle during swing occurred at heel strike. During stance, the hip angular velocity was predominantly an extension velocity and during swing primarily a flexion velocity. See Figure 2.

**Table 4:** Joint moments and powers during the stance phase of gait presented as mean ± s.d., normalized to body mass (Nm/kg) and (W/kg), with the *p*-value and Cohen’s d effect size, *p* (*d*). **Bold** indicates statistical significance.

	PRE	POST	*p* (*d*)
Peak dorsiflexion moment (Nm/kg)	0.28 ± 0.11	0.26 ± 0.09	0.127 (0.308)
Peak plantarflexion moment (Nm/kg)	−1.35 ± 0.15	−1.38 ± 0.014	0.067 (0.410)
Peak positive power generation (W/kg)	2.01 ± 0.57	2.34 ± 0.54	**0.001 (1.12)**
Peak negative power absorption (W/kg)	−0.86 ± 0.32	−0.87 ± 0.25	0.423 (0.051)
Peak knee extension moment (Nm/kg)	0.53 ± 0.24	0.59 ± 0.20	0.101 (0.346)
Peak knee flexion moment (Nm/kg)	−0.25 ± 0.09	−0.27 ± 0.07	0.087 (0.371)
Peak knee positive power generation (W/kg)	0.49 ± 0.24	0.56 ± 0.22	**0.044 (0.475)**
Peak knee negative power absorption (W/kg)	−1.35 ± 0.46	−1.29 ± 0.47	0.303 (0.137)
Peak hip flexion moment (Nm/kg)	0.53 ± 0.24	0.56 ± 0.25	0.208 (0.216)
Peak hip extension moment (Nm/kg)	−0.59 ± 0.17	−0.63 ± 0.16	0.094 (0.358)
Peak hip positive power generation (W/kg)	0.78 ± 0.23	0.87 ± 0.22	**0.026 (0.596)**
Peak hip negative power absorption (W/kg)	−0.47 ± 0.24	−0.56 ± 0.27	**0.020 (0.642)**

## Data Availability

The raw data supporting the conclusions of this article will be made available by the authors on request.
